# A Natural Flavonoid Glucoside, Icariin, Regulates Th17 and Alleviates Rheumatoid Arthritis in a Murine Model

**DOI:** 10.1155/2014/392062

**Published:** 2014-10-13

**Authors:** Liqun Chi, Wenyuan Gao, Xiangrong Shu, Xin Lu

**Affiliations:** ^1^School of Pharmaceutical Science and Technology, Tianjin University, Office A508, Building 24, 92 Weijin Road, Nankai District, Tianjin 300072, China; ^2^Haidian Maternal & Child Health Hospital of Beijing, China

## Abstract

Rheumatoid arthritis (RA) is a chronic autoimmune inflammatory disease that causes deformity of the joints and physical disability. Icariin, a natural flavonoid glucoside isolated from plants in the *Epimedium* family, has been proven to have various pharmacological activities. A recent study showed that icariin suppressed cartilage and bone degradation in mice of collagen-induced arthritis. However, the mechanism needs to be further investigated. In our current study, we found that icariin reduced the arthritis score and the incidence of arthritis compared with that in mice treated with water. Icariin inhibits the expression of various osteoclastogenic markers, such as *β*3 integrin, cathepsin K, and MMP9 *in vitro*. Icariin treatment in mice with CIA also resulted in less number of Th17 cells and decreased ratio of CD4^+^IL-17^+^ cells. The alleviated arthritis score and incidence of arthritis and reduced serum levels of IgG2a induced by icariin were abolished with additional IL-17 administration. Furthermore, icariin inhibited STAT3 activation in T cells and STAT3 inhibitor resulted in decreased IL-17 production and alleviated RA. In conclusion, icariin decreases Th17 cells and suppresses the production of IL-17, which contributes to the alleviated rheumatoid arthritis, through the inhibition of STAT3 activation.

## 1. Introduction

Rheumatoid arthritis (RA) is a chronic autoimmune inflammatory disease that causes deformity of the joints and physical disability [1]. The pain, swelling, stiffness, and tissue destruction that accompany inflammatory disease result from a cascade of events that are initiated and propagated by the production of inflammation cytokine [2]. Elevated levels of proinflammatory cytokines were demonstrated in RA patient sera and synovial fluids, such as IL-6, IL-8, TNF-α, IL-17, and IL-33 [3–5]. A number of these cytokines were not randomly present but formed a network or hierarchy which controlled their expression.

RA affects approximately 1% of the population, and up to 30% of RA patients become permanently work disabled within 3 years of diagnosis if they do not receive medical treatment [6, 7]. Although its etiology is unknown, our understanding of the cellular and molecular mechanisms that contribute to the pathogenesis in RA has allowed the development of new successful treatments for RA. However, there is an ongoing need for new therapeutics targeting different molecules because a substantial number of patients remain with active disease.

Icariin, a natural flavonoid glucoside isolated from plants in the* Epimedium* family, has been proven to have various pharmacological activities. Previous studies have indicated that icariin displayed positive effects on suppressing inflammatory and promoting cardiovascular function [8–11]. Furthermore, recent paper showed that icariin suppressed cartilage and bone degradation in mice of collagen-induced arthritis [12]. However, the mechanism needs to be further investigated.

In this study, we demonstrated a crucial role for icariin in regulating Th17 cells and alleviating rheumatoid arthritis. Icariin reduced the arthritis score and the incidence of arthritis compared with that in mice treated with water. Icariin inhibits osteoclastogenesis* in vivo* and* in vitro*. Furthermore, icariin decreases Th17 cells and suppresses the production of IL-17, which contributes to the alleviated rheumatoid arthritis, through inhibition of STAT3 activation.

## 2. Materials and Methods

### 2.1. Animals

Male C57BL/6 mice, all aged from 4 to 6 weeks, were purchased from the Center of Experimental Animals, Tianjin University, China. All experimental procedures were examined and approved by the Institutional Animal Care and Use Committee at Tianjin University.

### 2.2. Induction of Type II Collagen-Induced Arthritis (CIA) and Treatment with Icariin

Induction of CIA was conducted as described previously [13]. Beginning at the onset of CIA, the mice were dosed orally for 20 days with icariin (25 mg/kg) or an equal volume of water [12]. In some other experiments, mouse* in vivo* intraperitoneal injections included recombinant mouse IL-17 rmIL-17 (R&D Systems), 5 *μ*g per mouse for 20 days. Some animals were given 9 consecutive intraperitoneal injections of 0.5 mg/kg STAT3 inhibitor STA-21 (Santa Cruz Biotechnology) in saline, 3 times per week for 3 weeks [14].

### 2.3. Western Blotting

The protein levels were determined by Western blotting. Protein extracted from cells or tissue was separated on 10% SDS-polyacrylamide electrophoresis gels and transferred to nitrocellulose membranes (Pierce, Rockford, IL). After being blocked with 5% nonfat milk in TBS for 3 hours, the membranes were incubated with indicated primary antibodies (0.2 *μ*g/mL) at 4°C overnight, followed by incubation with HRP-conjugated secondary antibody (1 : 5000) for 3 hours. *β*-Actin was used as a loading control for comparison between samples.

### 2.4. Tissue Preparation and Histology

Mouse joint tissue was fixed in 4% paraformaldehyde, decalcified in EDTA bone decalcifier, and embedded in paraffin. Tissue sections (5 *μ*m) were prepared and stained with hematoxylin and eosin. Confocal microscopy was used to detect immunostaining for Th17 cells, as previously described [15]. To purify splenic CD4^+^ T cells, the splenocytes were isolated and incubated with CD4-coated magnetic beads and isolated using magnetic-activated cell sorting (MACS) separation columns (Miltenyi Biotec). MACS-sorted CD4^+^ cells were sorted to obtain naive cells by selecting for CD4^+^CD62L^high^CD25^−^CD44^low^ (>97% purity; DakoCytomation) or were purified using CD4^+^CD62L^+^ magnetic beads (Miltenyi Biotec).

### 2.5. Evaluation of* In Vitro* Osteoclastogenesis in Mouse Cells

Isolation of mouse bone marrow-derived monocyte/macrophage cells (BMM), differentiation of osteoclast precursor cells, tartrate-resistant acid phosphatase (TRAP) staining, and bone resorption analysis were performed as described previously [16].

### 2.6. Real-Time PCR

Total RNA was extracted from cultured cells or tissues using Trizol (Invitrogen, Carlsbad, CA) and reverse transcribed into cDNA using the PrimeScript RT reagent kit (Takara Biotechnology, Dalian, China) according to the manufacturer's instructions. mRNA levels of target genes were quantified using SYBR Green Master Mix (Takara Biotechnology, Dalian, China) with ABI PRISM 7900 sequence detector system (Applied Biosystems, Foster City, CA). Each reaction was performed in duplicate, and changes in relative gene expression normalized to 18sRNA levels were determined using the relative threshold cycle method.

### 2.7. Enzyme-Linked Immunosorbent Assay (ELISA)

The amounts of IL-17 and IgG2a were measured according to the manufacture's introduction.

### 2.8. Flow Cytometry Analysis

Isolated mononuclear cells from synovial tissue were cultured in 24-well plates in RPMI1640 medium supplemented with 10% FBS, 200 ng/mL phorbol myristate acetate (PMA, Sigma, St. Louis, MO), 400 ng/mL ionomycin, and brefeldin A (Sigma) for 4 h. The cells were harvested and stained with FITC-anti-human CD4 at 4°C for 30 min. After washing with PBS, the cells were fixed, permeabilized, and stained with APC-anti-IL-17 or PE-anti-IL-4 (eBioscience, San Diego, CA) at 4°C for 30 min. The frequencies of Th17 cells were analyzed using a FACS cytometer equipped with CellQuest software (BD Pharmingen).

### 2.9. Statistical Analysis

All data were presented as means ± SEM. The two-tailed Student's *t*-test was applied for statistical analysis with *P* < 0.05 being considered statistically significant. Data were analyzed using Prism software (GraphPad Software, Inc.).

## 3. Results

### 3.1. Icariin Suppresses Inflammatory Arthritis in Mice

**Figure 1:**
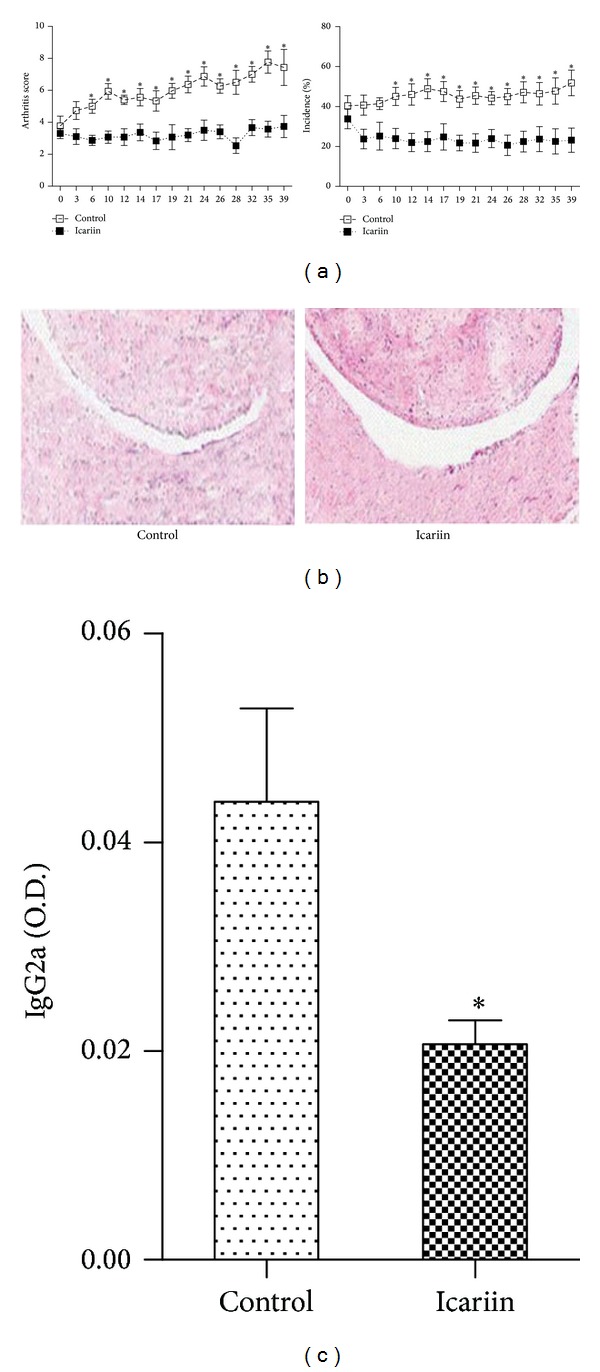
Icariin suppresses inflammatory arthritis in mice. (a) Arthritis score and incidence of arthritis in mice with CIA following treatment with icariin or control. (b) Histologic examination of the joints from mice with CIA in each group. (c) Levels of circulating IgG2a in the serum of mice with CIA. *P < 0.05 versus all other groups, *n* = 6.

We first investigated whether treatment with icariin would suppress the rheumatoid inflammation and joint destruction in mice with CIA. The results show that icariin reduced the arthritis score and the incidence of arthritis compared with that in mice treated with water (Figure 1(a)). Histological examination revealed that the joint of icariin-treated mice exhibited a lower degree of inflammation, as determined on day 40 after the treatment with icariin, when compared with the joint of water-treated mice (Figure 1(b)). In addition, the serum levels of IgG2a were significantly lower in mice treated with icariin (Figure 1(c)).

### 3.2. Icariin Decreases Th17 Cells and Represses IL-17 Production

**Figure 2:**
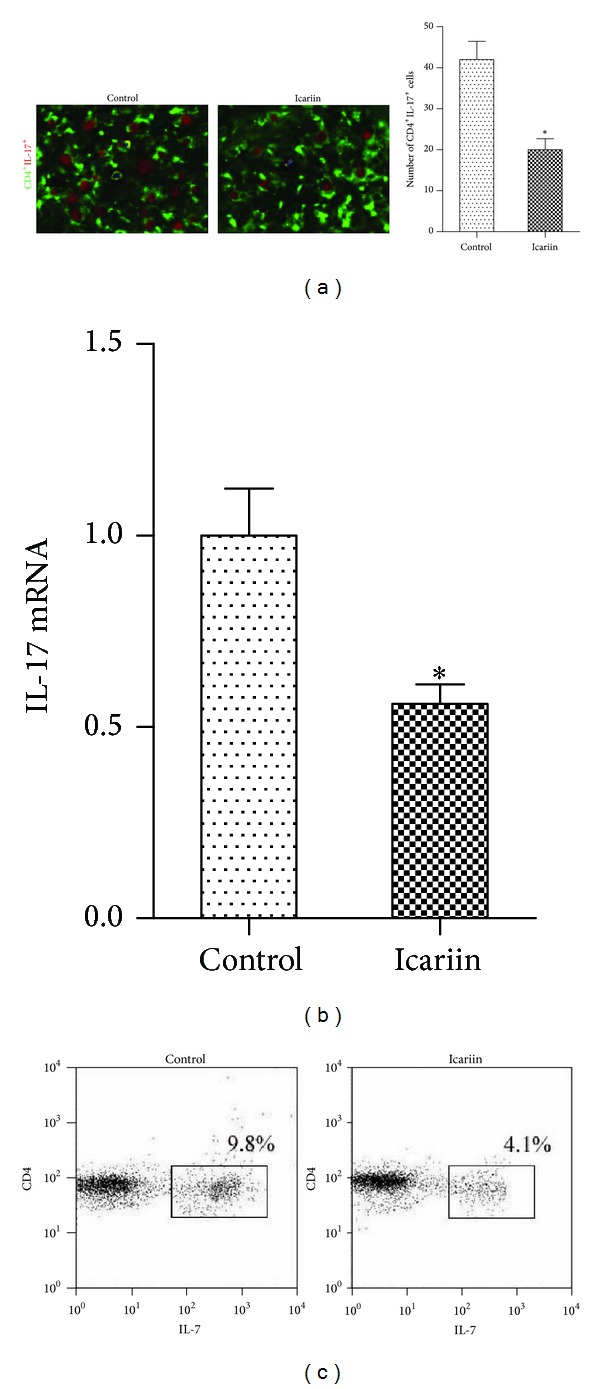
Icariin decreases Th17 cells and represses IL-17 production* in vivo*. (a) Samples of spleen tissue from mice treated with icariin or control were stained for CD4^+^IL-17^+^ T cells. (b) Expression of IL-17 mRNA was determined using real-time PCR in splenocytes from mice with CIA in each group. (c) Flow cytometry analysis of the synovial CD4^+^IL-17^+^ T cell subset in mice with CIA in each group. *P < 0.05 versus all other groups, *n* = 6.

We examined the numbers of CD4^+^IL-17^+^ Th17 cells in mouse spleens by performing confocal staining of the spleen tissue. The results indicate that the spleen tissue samples from mice treated with icariin showed decreased number of Th17 cells when comparing the spleen tissue samples from mice treated with control (Figure 2(a)). We also evaluated the expression of IL-17, which is the main cytokine that characterizes Th17 cells in the splenocytes using real-time PCR. The mice treated with icariin showed a decreased expression of IL-17 (Figure 2(b)). Next, we examined the synovial subset of CD4^+^IL-17^+^ Th17 cells using flow cytometry. The results showed that mice with CIA treated with icariin, as compared with mice treated with control, had profoundly decreased number of synovial Th17 cells (Figure 2(c)).

**Figure 3:**
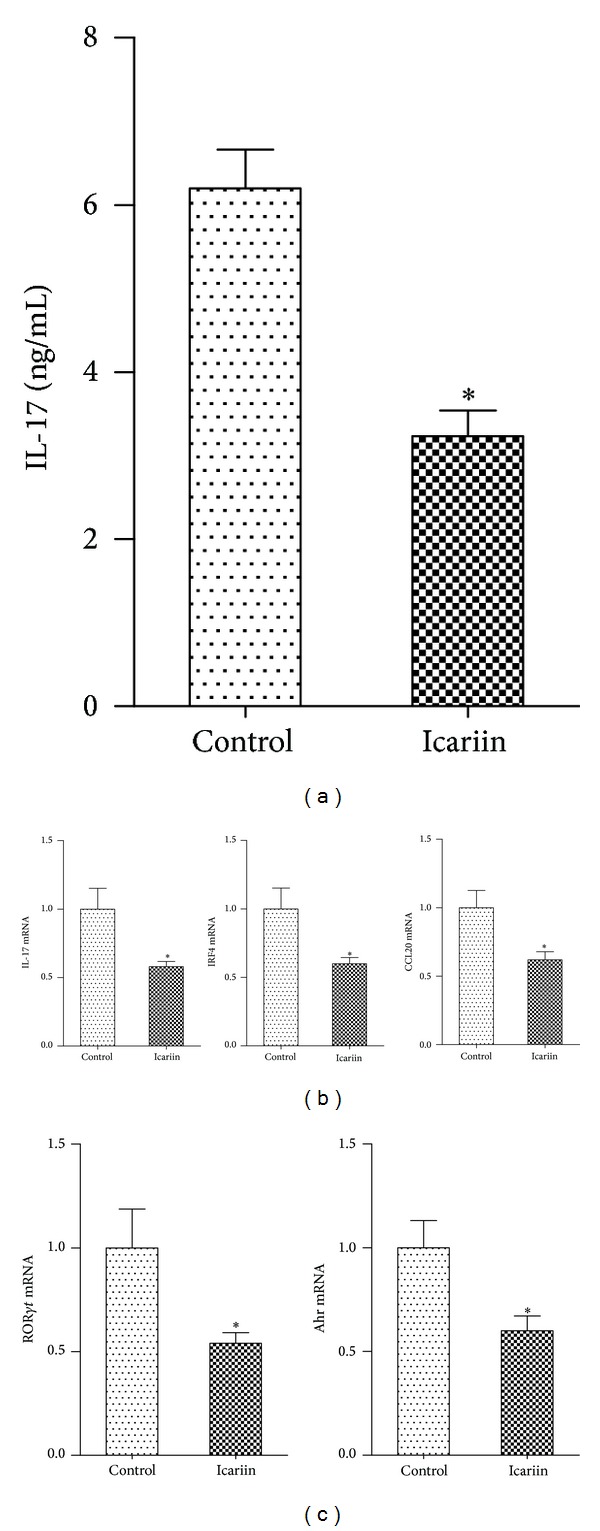
Icariin decreases Th17 cells and represses IL-17 production* in vitro*. CD4^+^ T cells from the spleens of mice were cultured under Th17 cell-inducing conditions with or without icariin (10 *μ*M/L). (a) After 72 hours of culture, expression of IL-17 mRNA in the cell was determined using PCR. (b) The concentration of IL-17 in culture supernatants of mouse serum was measured using ELISA. (c) The mRNA expression of molecules that are associated with Th17 cell differentiation, including retinoic acid receptor-related orphan nuclear receptor *γ*t (ROR*γ*t), aryl hydrocarbon receptor, interferon regulatory factor 4, and CCL20, was determined using real-time PCR. *P < 0.05 versus all other groups, *n* = 6.

We next examined the effect of icariin on Th17 cell differentiation* in vitro*. CD4^+^ T cells from mice were cultured in the presence of anti-CD3, TGF-*β*, and IL-6, with or without icariin, for 72 hours. We found that* in vitro* treatment with icariin substantially decreased the levels of IL-17 in the culture supernatants of mouse serum and the expression of IL-17 mRNA (Figures 3(a)-3(b)). Treatment with icariin also decreased the mRNA levels of retinoic acid receptor-related orphan nuclear receptor *γ*t (ROR*γ*t), aryl hydrocarbon receptor, interferon regulatory factor 4, and CCL20, all of which are factors implicated in Th17 cell differentiation (Figure 3(c)).

### 3.3. Icariin Inhibits Osteoclastogenesis in Mice

**Figure 4:**
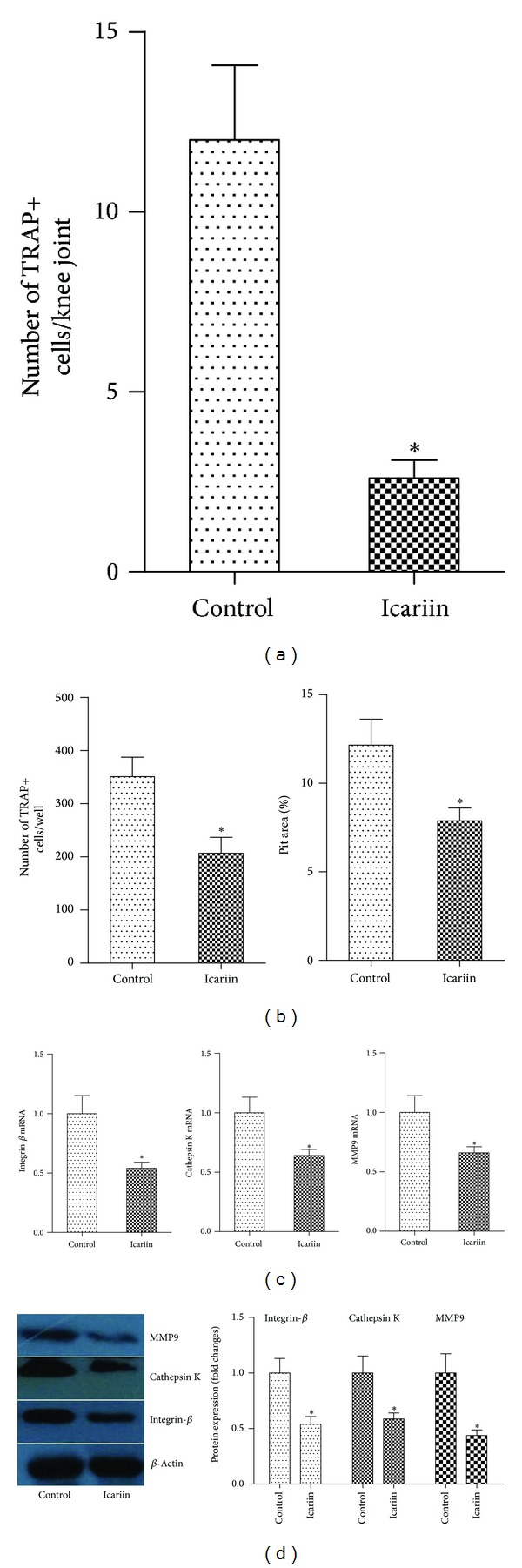
Icariin inhibits osteoclastogenesis in mice. (a) The number of TRAP^+^ cells in synovial tissue sections from the joints of mice with CIA. (b) Osteoclast precursor cells from bone marrow-derived monocytes/macrophage (BMM) cells of normal mice were further cultured in the presence of M-CSF (10 ng/mL) together with RANKL (50 ng/mL) with or without icariin. The number of TRAP^+^ cells and percentage of pit area were determined. (c) The mRNA expression of various osteoclastogenic markers was analyzed using real-time PCR. (d) The protein level of various osteoclastogenic markers was analyzed using Western blot. *P < 0.05 versus all other groups. *n* = 6.

Osteoclasts are primarily involved in the bone destruction of RA. RANKL is the key osteoclastogenic molecule expressed by osteoclastogenesis-supporting cells [17, 18]. To examine the effect of icariin on osteoclast formation, we performed TRAP staining on the tissue section from the joints. The results showed that the joint tissue of icariin-treated mice showed a markedly reduced formation of osteoclasts compared with the joint tissue of the control mice (Figure 4(a)).

We next examined whether icariin inhibits osteoclast formation* in vitro*. BMM cells were prepared from normal mice and then stimulated by M-CSF together with RANKL. The addition of icariin to the cell culture markedly reduced TRAP-positive cells (Figure 4(b)). The mRNA and protein levels of various osteoclastogenic markers, such as *β*3 integrin, cathepsin K, and MMP9, were also significantly decreased by the addition of icariin (Figures 4(c)-4(d)).

### 3.4. Icariin Suppresses Inflammatory Arthritis Partly Dependent on Inhibited IL-17 Production

**Figure 5:**
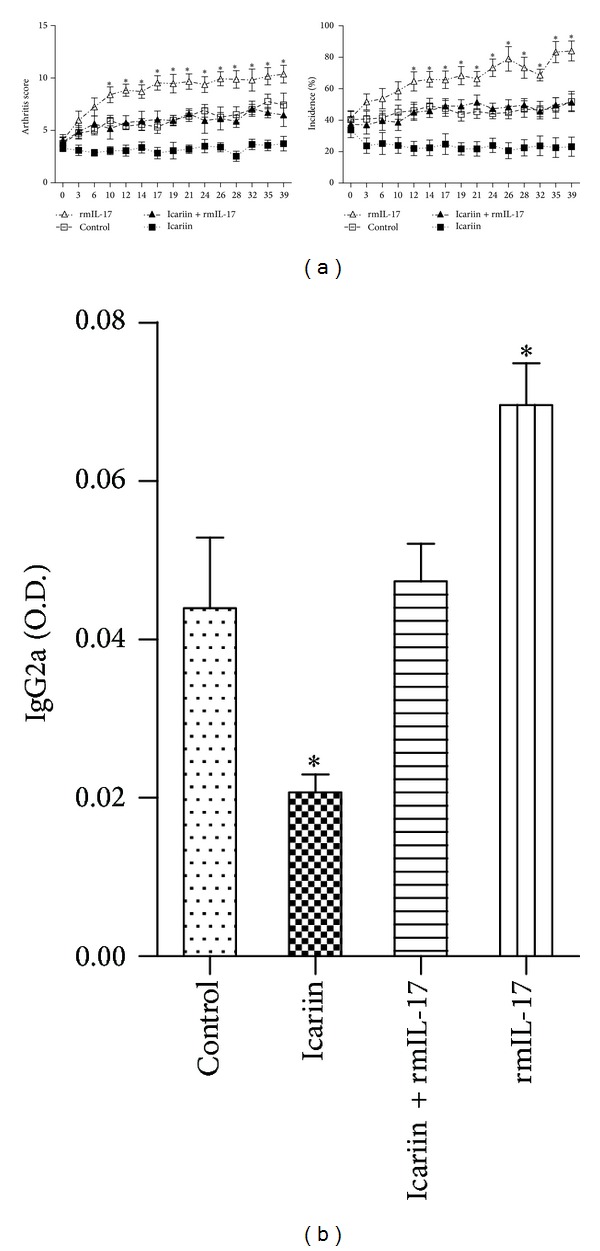
Icariin suppresses inflammatory arthritis partly dependent on inhibited IL-17 production, indicating arthritis score and incidence of arthritis in mice with CIA in the control, icariin, icariin + rmIL-17, and rmIL-17 groups. *P < 0.05 versus all other groups, *n* = 6.

Elevated IL-17 is associated with RA. To demonstrate that IL-17 inhibition is the major mediator of the suppressed inflammatory response in icariin-treated mice with CIA, icariin-treated mice were administered with recombinant mouse IL-17 (rmIL-17). Results show that the alleviated arthritis score and incidence of arthritis and reduced serum levels of IgG2a induced by icariin were all abolished with additional IL-17 administration (Figures 5(a)-5(b)).

### 3.5. Icariin Inhibited STAT3 Activation in T Cells* In Vivo *and* In Vitro*

**Figure 6:**
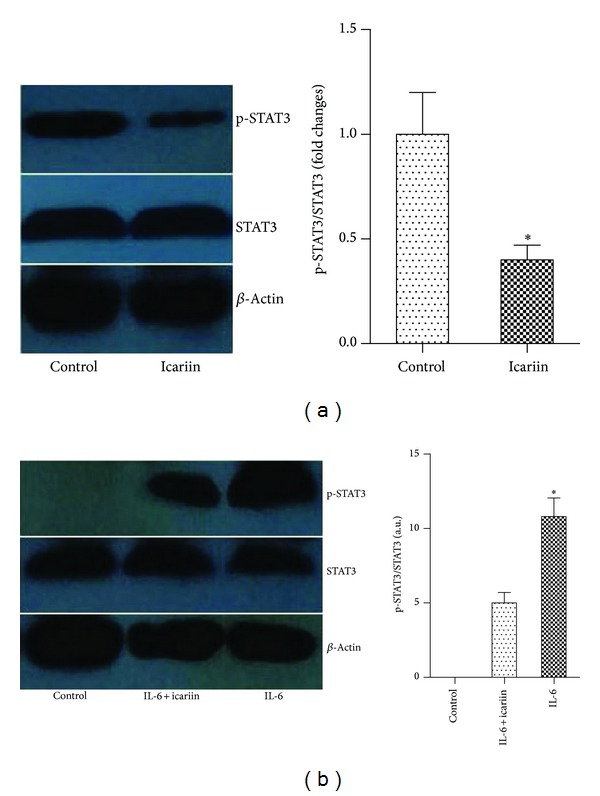
Icariin inhibited STAT3 activation in T cells* in vivo* and* in vitro*. (a) Synovial p-STAT3 protein level was analyzed using Western blotting. (b) Naive T cells from mice were treated with 3 *μ*M icariin for 24 hours, followed by stimulation of IL-6 (25 ng/mL) for 30 min. Protein levels were assessed using Western blot. *P < 0.05 versus all other groups, *n* = 6.

We first investigated the effect of icariin on STAT3 phosphorylation in the joint tissue of mice with CIA. The Western blotting indicated that icariin inhibited STAT3 phosphorylation in synovial tissue of mice with CIA (Figure 6(a)). Next,* in vitro* tests were performed using T cells derived from naive lymph node cells of C57BL/6 mice. The phosphorylation of STAT3 upon IL-6 stimulation, which is typically associated with Th17 differentiation, was also inhibited by icariin (Figure 6(b)).

### 3.6. STAT3 Inhibitor Suppresses IL-17 Production in Mice with CIA

**Figure 7:**
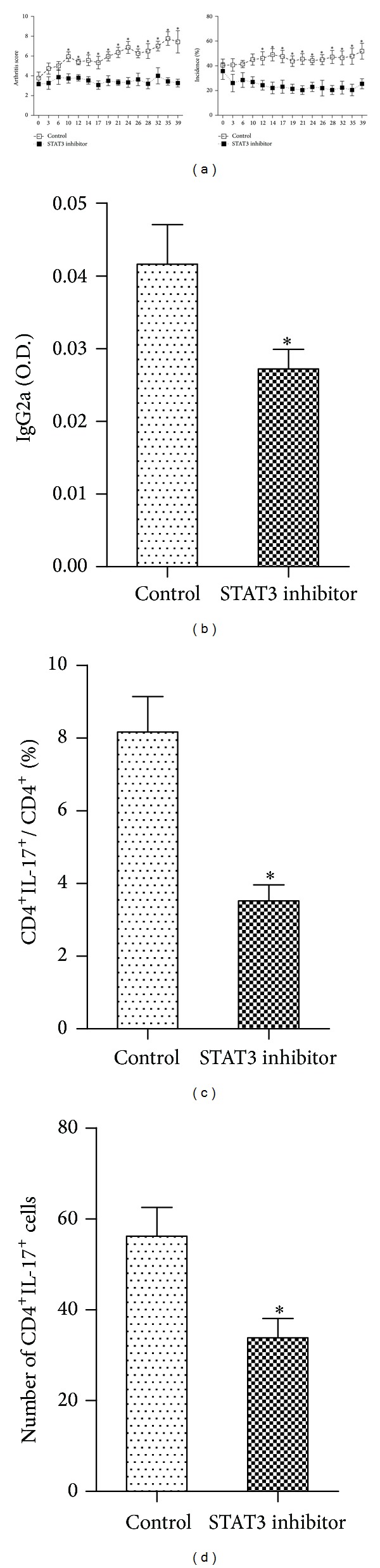
STAT3 inhibitor suppresses IL-17 production in mice with CIA. (a) Arthritis score and incidence of arthritis in mice with CIA following treatment with STAT3 inhibitor or control. (b) Levels of circulating IgG2a in the serum of mice with CIA. (c) Flow cytometry analysis of the synovial CD4^+^IL-17^+^ T cell subset in mice with CIA. (d) Samples of spleen tissue from mice treated with STAT3 inhibitor or control were stained for CD4^+^IL-17^+^ T cells. *P < 0.05 versus all other groups, *n* = 6.

We further investigated whether STAT3 inhibitor would suppress IL-17 production in mice with CIA. The result shows that inhibition of STAT3 reduced the arthritis score and the incidence of arthritis compared with that in mice treated with control (Figure 7(a)). The serum levels of IgG2a were markedly lower in mice treated with STAT3 inhibitor (Figure 7(b)). Furthermore, STAT3 inhibitor also significantly decreased IL-17 expression in mice with CIA (Figures 7(c)-7(d)).

## 4. Discussion

Our current study demonstrated a crucial role for icariin in regulating Th17 cells and alleviating rheumatoid arthritis. Icariin reduced the arthritis score and the incidence of arthritis compared with that in mice treated with water. Icariin inhibits osteoclastogenesis* in vivo* and* in vitro*. Furthermore, icariin decreases Th17 cells and suppresses the production of IL-17, which contributes to the alleviated rheumatoid arthritis through the inhibition of STAT3 activation.

Cathepsin K, a cysteine protease active at acidic pH, is the most abundant collagenase in osteoclasts, and its excessive expression in the bone and cartilage has been found to result in osteoporosis and arthritis, respectively. Previous paper has indicated that overexpression of cathepsin K gene resulted in progressive synovitis and aggravated destruction of the articular cartilage and the bone [19, 20]. Icariin, a cathepsin K inhibitor [12], reduced the formation of osteoclasts* in vivo* and* in vitro* in our current study. This was consistent with previous report indicating that inhibition of cathepsin K reduced bone and cartilage degradation evoked by collagen-induced arthritis in mice [21].

One of the most significant characteristics of RA is the intensive inflammation that is out of control. Managing to control the inflammation could prevent the disease progression, which would be the optimal strategy for RA therapy. One direct strategy is targeting the pathogenic inflammatory cytokines. Interleukin- (IL-) 17A is a member of the IL-17 family, which includes six structurally related isoforms: IL-17A, IL-17B, IL-17C, IL-17D, IL-17E, and IL-17F. IL-17A was secreted by different cells, including Th17 cells, *γ* *δ* T cells, NK cells, NKT cells, and neutrophils. IL-17A is a critical mediator of neutrophil recruitment and migration through the induction of granulopoiesis and neutrophil chemokines. IL-6 is a key regulator of IL-17 production, whereas it inhibits the regulatory T cells [22]. IL-23 plays an essential role in various inflammatory diseases, which can promote IL-17 production as well as IL-22 and IL-17 produced by Th17 cells. IL-17A, which synergizes with TNF-α to promote the activation of fibroblasts and chondrocytes, is currently being targeted in clinical trials [23]. The results in our study indicated suppressed Th17 cells differentiation* in vivo* and* in vitro* with treatment of icariin. The alleviated arthritis score and incidence of arthritis induced by icariin were abolished with additional IL-17 administration. These suggest that icariin alleviated RA at least partly dependent on inhibited IL-17 production.

Phosphorylation of STAT3 is considered critical for constraining IL-17 production [24, 25]. IL-17 involved in the pathophysiology of inflammatory diseases required specific cytokines and transcription factors for their differentiation. STAT3 is activated and phosphorylated by JAK. Activated STAT-3 translocates into the nucleus and then promotes the transcription of ROR*γ*t, the essential transcription factor of Th17 cells. It also regulates the expression of IL-17, IL-21, and IL-23R, all of which are important in the effector function of Th17 cells. Moreover, the excessive expression of IL-17 activated the STAT3 signaling pathway. Therefore, an interconnected loop formed RA inflammatory factors, IL-17, STAT3 signaling pathway, and IL-17-related cytokines, which could influence each other and promote the development of RA. Thus, mice that lack STAT-3 in CD4^+^ T cells are unable to generate Th17 cells and are resistant to animal models of autoimmunity [26, 27]. In our current study, icariin inhibited STAT3 activation* in vivo* and* in vitro*. Furthermore, STAT3 inhibitor significantly suppressed IL-17 production and attenuated the arthritis score and the incidence of arthritis. All of these demonstrated that icariin alleviated RA and suppressed Th17 differentiation at least partly through the inhibition of STAT3 activation.

In summary, our study provides evidence that icariin regulates Th17 cells and alleviates rheumatoid arthritis. Icariin reduced the arthritis score and the incidence of arthritis compared with that in mice treated with water. Icariin inhibits osteoclastogenesis* in vivo* and* in vitro*. Furthermore, icariin decreases Th17 cells and represses the production of IL-17, which contributes to the alleviated rheumatoid arthritis, through the inhibition of STAT3 activation. Our study identifies a previously unknown immune role for icariin in Th17 differentiation and RA. Although further investigations are needed to fully clarify the precise molecular and cellular mechanism involved in immunoregulation, icariin may be a novel target to attenuate RA.
